# *Lagerstroemia ovalifolia* Exerts Anti-Inflammatory Effects in Mice of LPSInduced ALI via Downregulating of MAPK and NF-κB Activation

**DOI:** 10.4014/jmb.2107.07023

**Published:** 2021-09-03

**Authors:** Jae-Hong Min, Seong-Man Kim, JI-Won Park, Nam Hoon Kwon, Soo Hyeon Goo, Ngatinem, Sri Ningsih, Jin-Hyub Paik, Sangho Choi, Sei-Ryang Oh, Sang-Bae Han, Kyung-Seop Ahn, Jae-Won Lee

**Affiliations:** 1 Natural Medicine Research Center, Korea Research Institute of Bioscience and Biotechnology, Cheongju 28116, Republic of Korea; 2 Starch Technology Center, Agency for the Assessment and Application Technology, Lampung 34161, Indonesia; 3 Center for Pharmaceutical and Medical Technology, Agency for the Assessment and Application of Technology, LAPTIAB Building 611, Puspiptek, Serpong, Tangerang-Selatan 15314, Indonesia; 4 International Biological Material Research Center, Korea Research Institute of Bioscience and Biotechnology, Daejeon 34141, Republic of Korea; 5 College of Pharmacy, Chungbuk National University, Cheongju 28160, Republic of Korea

**Keywords:** Acute lung injury, *Lagerstroemia ovalifolia* leaf extract, cytokines, NF-κB, HO-1

## Abstract

*Lagerstroemia ovalifolia* Teijsm. & Binn. (LO) (crape myrtle) has reportedly been used as traditional herbal medicine (THM) in Java, Indonesia. Our previous study revealed that the LO leaf extract (LOLE) exerted anti-inflammatory effects on lipopolysaccharide (LPS)-stimulated RAW264.7 macrophages. Based on this finding, the current study aimed to evaluate the protective effects of LOLE in a mouse model of LPS-induced acute lung injury (ALI). The results showed that treatment with LPS enhanced the inflammatory cell influx into the lungs and increased the number of macrophages and the secretion of the inflammatory cytokines in the bronchoalveolar lavage fluid (BALF) of mice. However, these effects were notably abrogated with LOLE pretreatment. Furthermore, the increase of inducible nitric oxide synthase (iNOS), cyclooxygenase-2 (COX-2) and monocyte chemoattractant protein-1 (MCP-1) expression in the lung tissues of mice with ALI was also reversed by LOLE. In addition, LOLE significantly suppressed the LPS-induced activation of the MAPK/NF-κB signaling pathway and led to heme oxygenase-1 (HO-1) induction in the lungs. Additionally, in vitro experiments showed that LOLE enhanced the expression of HO-1 in RAW264.7 macrophages. The aforementioned findings collectively indicate that LOLE exerts an ameliorative effect on inflammatory response in the airway of ALI mice.

## Introduction

Acute lung injury (ALI), which may be induced by numerous factors, such as infection, is closely related to significant mortality [1]. Accumulating evidence has supported the importance of macrophage recruitment and macrophage-derived molecules (cytokines, chemokines and mediators) in the development of ALI [2]. TNF-α and IL-6 upregulation could promote airway inflammatory responses in ALI [3, 4]. Meanwhile, upregulation of inducible nitric oxide synthases (iNOS) was also confirmed in both clinical and pre-clinical studies on ALI [5] and iNOS-induced nitric oxide (NO) expression contributed to the development of ALI via generating peroxynitrite [6]. Another study demonstrated that the increased expression of cyclooxygenase-2 (COX-2) was associated with edema and pain sensitization in ALI [7]. Furthermore, macrophage-derived monocyte chemoattractant protein 1 (MCP-1) could promote the inflammatory responses during the pathogenesis of ALI via affecting the recruitment of inflammatory cells, such as monocytes and macrophages [8]. It has been reported that MAPK/NF-κB signaling pathways lead to inflammatory responses in ALI via enhancing the expression of inflammatory cytokines, chemokines and mediators [9-12]. In ALI, alleviated airway inflammation is characterized by the expression of antioxidant proteins, such as heme oxygenase-1 (HO-1) [13-15].

Plant extracts have been shown to exert protective effects against acute or chronic inflammatory lung diseases [16-18]. *Lagerstroemia ovalifolia* Teijsm. & Binn. (LO), or crape myrtle, is a member of the Lythraceae family distributed in Java and has been used there as traditional herbal medicine (THM) [19, 20]. A previous study by Park *et al*. showed that LO leaf extract (LOLE) exerted anti-inflammatory effects οn the lipopolysaccharide (LPS)-induced inflammatory responses in RAW264.7 macrophages [19]. In that study, treatment with LOLE significantly decreased cytokines/mediators and MAPK/NF-κB activation, thus supporting its ameliorative effect on ALI. Therefore, in this work we explored the protective effects of LOLE in mice with lipopolysaccharide (LPS)-induced ALI.

## Materials and Methods

### LOLE Preparation

LO leaves were collected from the Pangandaran Nature Reserve, West Java, Indonesia, and formally identified by staff of the Center for Pharmaceutical and Medical Technology (PTFM). A voucher specimen (KRIB 0038535) was deposited in the herbarium of the Korea Research Institute of Bioscience and Biotechnology (KRIBB), respectively. A total of 100 g of powder-dried leaves of LO was extracted with 2 L of methanol with agitation for 1 h and left overnight at room temperature. Then, the methanol extract was filtered and extraction was repeated twice. The collected filtrate was concentrated using a rotary evaporator (Rotavapor 4000; Heidolph) and then a semisolid mass was obtained. The *L. ovalifolia* Teijsm. & Binn. leaf extract (LOLE) was kept in a sealed dark-glass container until further use [19].

### Cell Culture

Murine macrophage cell line RAW264.7 cells were purchased from ATCC and maintained in DMEM (HyClone; Cytiva) supplemented with 10% FBS and 1% antibiotic and antimycotic reagent at 37°C in a humidified incubator with 5% CO_2_. To detect the expression of HO-1, cells were seeded into 6-well plates at a density of 5 × 10^5^ cells/well and were then treated with 2.5, 5, or 10 μg/ml LOLE for 16 h in the absence or presence of LPS (500 ng/ml).

### LPS-induced Mouse Model of ALI

C57BL/6 mice (*n* = 30; male; 6 weeks old; 18 ± 1 g) were obtained from Koatech Co., Ltd. The experimental procedure was approved by the Institutional Animal Care and Use Committee of the KRIBB (KRIBB-AEC-21111). To establish ALI, mice were treated with LPS and the indicated drugs as previously described [13]. Five experimental groups were established for in vivo study as follows: the normal control group (NC group), LPS group (lipopolysaccharide only), LPS+DEX group [LPS + oral gavage (o.g.) of 1 mg/kg DEX], LPS+LOLE 10 group (lipopolysaccharide + o.g. of 10 mg/kg LOLE) and LPS+LOLE 20 group (lipopolysaccharide + o.g. 20 mg/kg LOLE). Subsequently, on days 1-3, mice were orally administered LOLE or dexamethasone (DEX) as positive control. On day 3, at 1 h following the last treatment with LOLE or DEX, mice were intranasally administered LPS (0.5 mg/kg in 40 μl PBS).

### Measurement of Macrophage Number and Cytokine Secretion in BALF

To measure macrophage numbers and TNF-α and IL-6 secretion, BALF was isolated as previously described [3, 21]. Mice were anesthetized by intraperitoneal injection (i.p.) of 30-50 mg/kg Zoletil 50 (Virbac Korea Co., Ltd.) and 5-10 mg/kg xylazine (Bayer) on day 5, as previously described [3]. Subsequently, 700 μl PBS was infused into the trachea and BALF was collected. To morphologically distinguish macrophages, cells in BALF were seeded on glass slides and stained with a Diff-Quik Stain Kit (Sysmex Crop. Japan). Then, the number of macrophages was calculated by light microscope observation (magnification, ×400). The secretion levels of cytokines in BALF were measured using the corresponding ELISA kits.

### Western Blotting

Mice were sacrificed by cervical dislocation prior to the collection of lung tissues. Lung tissue and cell culture lysates were prepared as previously described [19, 22] and a BCA assay was performed to determine the concentrations of protein. Subsequently, protein separation was performed by SDS-PAGE and separated proteins were transferred onto PVDF membranes. The membranes were then blocked with 5% skim milk and were maintained with primary antibodies against phosphorylated (p)-ERK (9101), ERK (9102), p-p38 (9211), p-JNK (4668), JNK (9252), p-p65 (3033), p65 (8242), and p-IκBα (2859, dilutions, 1/1,000; Cell Signaling Technology, Inc.), MCP-1 (17040), IκBα (15132), HO-1 (27338, dilutions, 1/1,000; Invitrogen Crop., USA), p38 (sc-7149), β-actin (sc-69879, dilutions, 1/1,000; Santa Cruz Biotechnology, Inc., USA) and iNOS (905-431, dilutions, 1:1,000; Enzo Life Sciences, Inc., USA). The membranes were then washed with TBST and incubated with the corresponding secondary antibodies. Finally, the blots were visualized using an ECL kit.

### Histological Analysis

For the detection of histological changes, the collection of lung was performed on day 5, and the 10% formalin-fixed lung tissue was embedded in paraffin. Subsequently, the paraffin-embedded lung tissue was cut into 4-μm sections using a microtome. Then, sections were stained with a hematoxylin and eosin (H&E) staining solution.

### Statistical Analysis

Values are presented as the mean ± SD. One-way ANOVA with Tukey’s multiple comparison test was applied to reveal significant differences among multiple groups (SPSS 20.0 IBM Corp., USA). *p* < 0.05 was considered to indicate a statistically significant difference.

## Results

### Effect of LOLE on Inflammatory Cell Recruitment and Cytokine Secretion in Mice with ALI

**Fig. 1:**
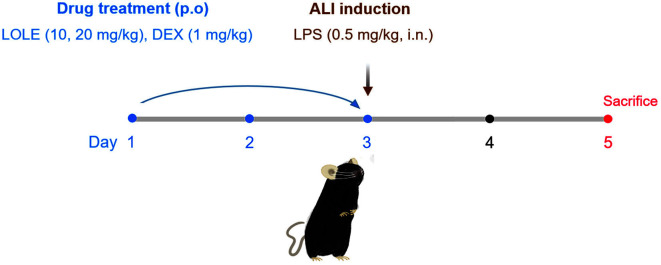
Experimental procedure for the establishment of ALI injury mouse model and treatment of mice with LOLE or DEX. From days 1-3, oral administration of LOLE (10 or 20 mg/kg) or DEX (1 mg/kg) to mice was performed. On day 3, at 1 h following the last treatment with LOLE or DEX, mice were intranasally administrated with lipopolysaccharide (0.5 mg/kg in 40 μl PBS). On day 5, the collection of BALF and lung was performed. LOLE, *Lagerstroemia ovalifolia* Teijsm. & Binn. leaf extract; DEX, dexamethasone.

**Fig. 2:**
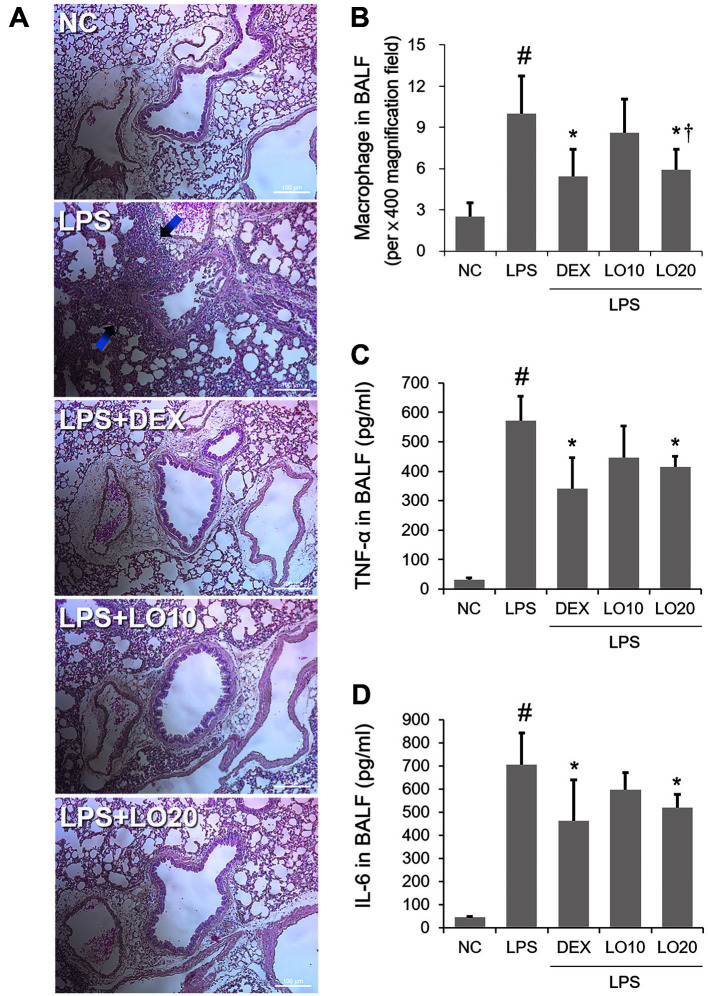
LOLE decreases inflammatory cells influx and cytokine secretion in the lungs of ALI mice. (**A**) H&E staining was carried out to detect inflammatory cell influx (magnification, x100; scale bar, 100 μm). (**B**) Diff-Quik staining was carried out to measure the number of macrophages in BALF. (**C**) TNF-α and IL-6 levels in BALF were detected by ELISA kits. Data are expressed as the mean ± SD. ^#^*p* < 0.05 compared to normal control (NC) group; *p < 0.05 compared to LPS group; ^†^*p* < 0.05 compared to LO 10 mg/kg group. LO, *Lagerstroemia ovalifolia* Teijsm. & Binn. leaf extract (LOLE); LPS, lipopolysaccharide; BALF, bronchoalveolar lavage fluid.

Airway inflammation was achieved by intranasal administration of LPS (Fig. 1). The H&E staining results showed an outstanding existence of inflammatory cells near the airway in the lungs of ALI mice (Fig. 2A). Interestingly, this effect was ameliorated by LOLE pretreatment. Diff-Quik staining results revealed that the notable increase of macrophages in the BALF of mice with ALI was decreased following pretreatment with LOLE (Fig. 2B). In addition, the ELISA results showed that TNF-α and IL-6 secretion was significantly increased in the BALF of mice with ALI, while this effect was also reversed by LOLE (Figs. 2C and 2D).

### Effect of LOLE on iNOS, COX-2 and MCP-1 Expression in Mice with ALI

**Fig. 3:**
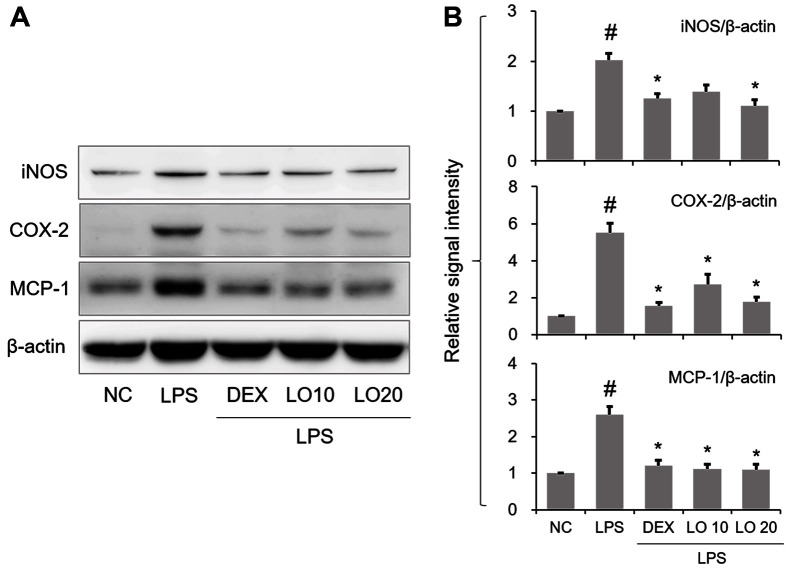
LOLE downregulates iNOS, COX-2 and MCP-1 in the lung tissues of ALI mice. (**A**) The protein expression levels of iNOS, COX-2 and MCP-1 were determined by Western blot analysis. (**B**) The expression levels of iNOS, COX-2 and MCP-1 were quantified by densitometric analysis. Data are expressed as the mean ± SD. ^#^*p* < 0.05 compared to NC group; *p < 0.05 compared to LPS group.

Western blot analysis showed that iNOS, COX-2 and MCP-1 were upregulated in the lungs of mice with ALI compared with the NC group (Figs. 3A and 3B). However, LOLE inhibited the expression of these proteins compared to ALI group.

### Inhibitory Effect of LOLE on MAPK Activation in Mice with ALI

**Fig. 4:**
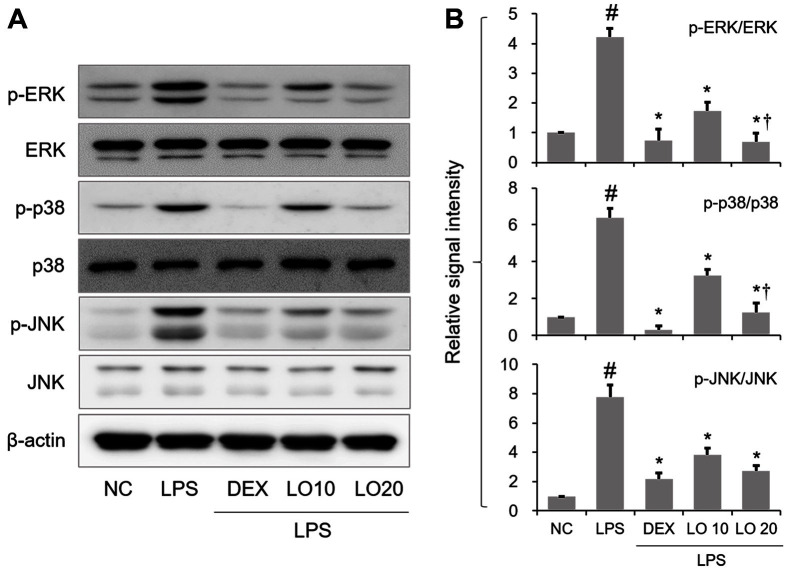
LOLE leads to MAPK inactivation in lung of ALI mice. (**A**) The phosphorylation of MAPK protein was determined by Western blot analysis. (**B**) The protein expression levels of p-ERK, p-p38 and p-JNK were quantified using densitometric analysis. Data are expressed as the mean ± SD. ^#^*p* < 0.05 compared to NC group; *p < 0.05 compared to LPS group; ^†^*p* < 0.05 compared to LO 10 mg/kg group.

Subsequently, MAPK (ERK, p38 and JNK) phosphorylation was determined by Western blot analysis. The levels of p-ERK was markedly upregulated in the lung tissues of mice with ALI. However, their expression was notably downregulated following LOLE pretreatment. Consistent with the previous finding, LOLE reduced the LPS-induced phosphorylation of p38 and JNK (Figs. 4A and 4B).

### Inhibitory Effect of LOLE on NF-κB Activation in Mice with ALI

**Fig. 5:**
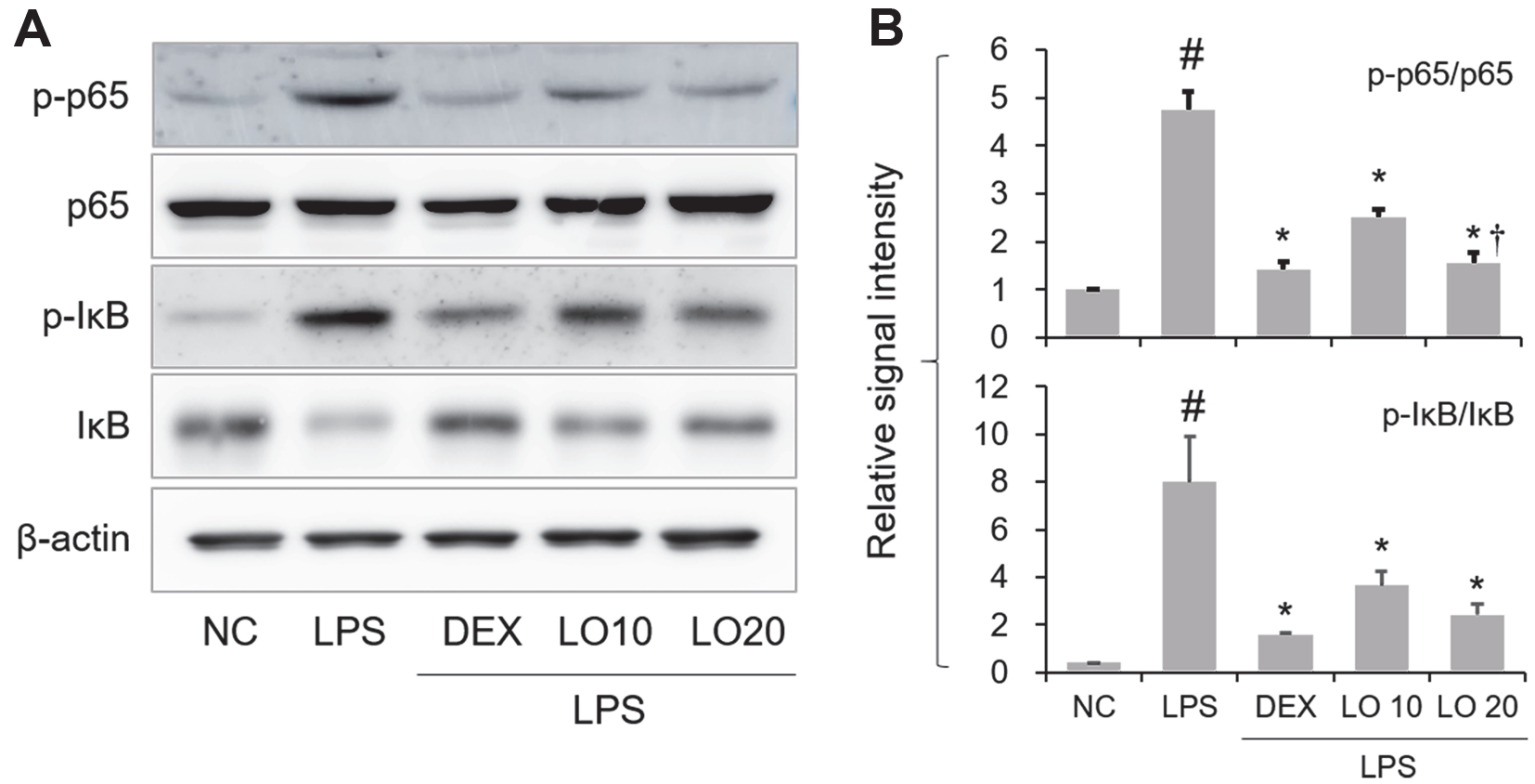
LOLE leads to NF-κB inactivation in lung of ALI mice. (**A**) NF-κB activation was determined by Western blotting. (**B**) The quantification of p-NF-κB and p-IκB was performed using densitometric analysis. Data are expressed as the means ± SD. ^#^*p* < 0.05 compared to NC group; *p < 0.05 compared to LPS group; ^†^*p* < 0.05 compared to LO 10 mg/kg group.

Based on the inhibitory effect of LOLE on inflammatory molecules, the regulatory effect of LOLE on NF-κB activation was assessed by Western blot analysis. As shown in Figs. 5A and 5B, phosphorylation of NF-κB p65 was notably upregulated in lung of ALI mice compared to NC group, whereas this trend was attenuated by LOLE pretreatment. Consistently, pretreatment of mice with LOLE significantly restored the LPS-induced upregulation of IκB phosphorylation compared with the LPS group.

### Effect of LOLE on HO-1 Induction in Mice and RAW264.7 Macrophages

**Fig. 6:**
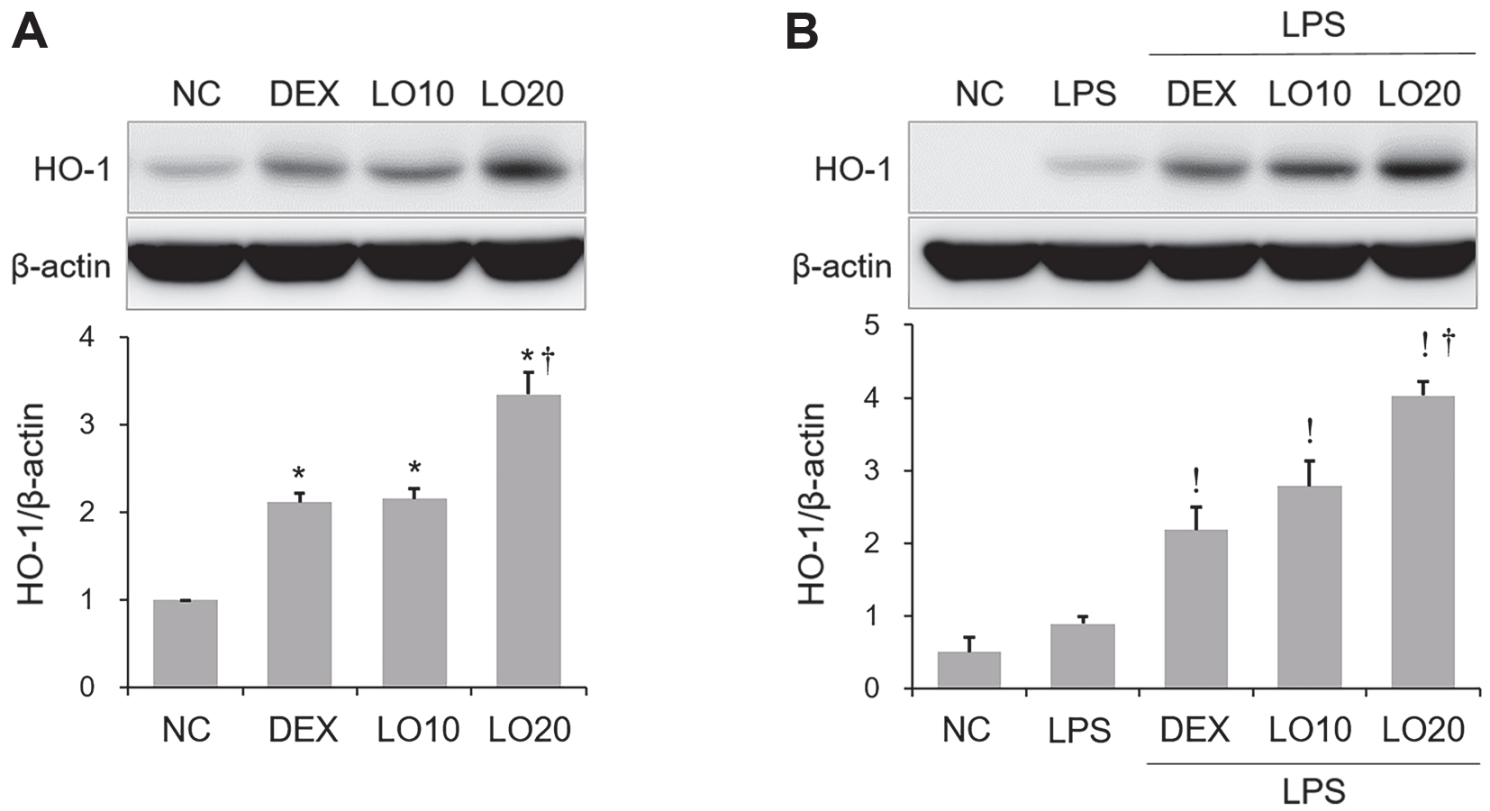
LOLE induces HO-1 expression in lung of mice or ALI mice. The protein expression level of HO-1 was determined by Western blotting. HO-1 expression was quantified using densitometric analysis. Data are expressed as the mean ± SD. *p < 0.05 compared to NC group; ^†^*p* < 0.05 compared to LO 10 mg/kg group; ^!^*p* < 0.05 compared to LPS group. HO-1, heme oxygenase-1.

**Fig. 7:**
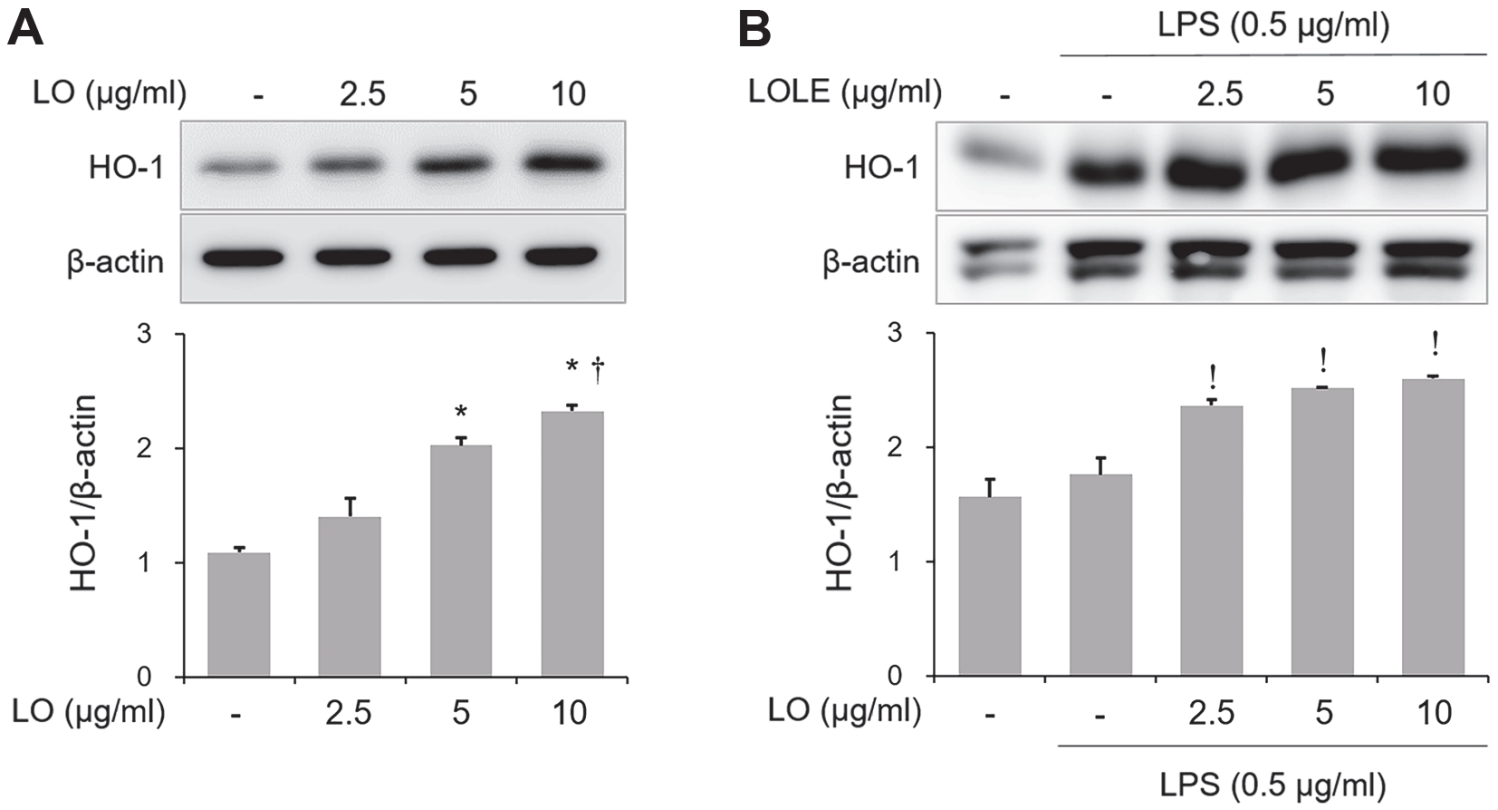
LOLE increases HO-1 expression in RAW264.7 cells or activated RAW264.7 cells. HO-1 induction was determined in RAW264.7 cells by Western blotting. The quantification of HO-1 was performed using densitometric analysis. Data are expressed as the mean ± SD. *p < 0.05 compared to NC group; ^†^*p* < 0.05 compared to LO 5 μg/ml group; ^!^*p* < 0.05 compared to LPS group.

Previous studies showed that the activation of HO-1 could alleviate inflammation in an experimental model of ALI [13, 23]. Therefore, in the current study we explored whether LOLE could upregulate HO-1 in murine lung tissues and in RAW264.7 cells. The results showed that LOLE treatment induced HO-1 expression in lung of mice or LPS-exposed ALI mice (Figs. 6A and 6B). Consistently, LOLE could upregulate HO-1 in RAW264.7 cells or LPS-stimulated RAW264.7 cells (Figs. 7A and 7B).

## Discussion

Macrophage-derived TNF-α and IL-6 are considered as the most important markers in ALI [2]. As described above, this cell-derived iNOS and COX-2 were associated with oxidation and pain, respectively, in ALI [6, 7]. In addition, the hyper-inflammation in ALI was related to MCP-1 in macrophages [8]. Therefore, regulating macrophage influx and the expression of cell-derived molecules played an important role in ameliorating LPS-induced ALI [24]. The current study demonstrated that LOLE exerted an anti-inflammatory effect on LPS-induced airway inflammation via attenuating macrophage influx and the expression of the inflammatory molecules (TNF-α, IL-6, iNOS, COX-2 and MCP-1). These findings suggested that LOLE may exert an inhibitory effect on pulmonary inflammation in endotoxin-induced ALI.

Accumulating evidence has highlighted the MAPK and NF-κB signaling pathways in ALI [25-28]. A previous study demonstrated that LOLE suppresses MAPK/NF-κB activation and TNF-α, IL-6, iNOS and COX-2 expression in activated RAW264.7 macrophages [19]. Herein, the inhibitory effect of those molecules was also verified in mice with ALI. Based on these findings, we investigated whether LOLE could also regulate MAPK/NF-κB activation in vivo. As expected, the regulatory effect of LOLE on the activation of MAPK/NF-κB was confirmed. These results indicated that the ameliorative effect of LOLE on airway inflammation could be associated with the regulation of MAPK/NF-κB signaling activation.

The decreased expression of inflammatory cytokines/chemokines and the suppression of MAPK/NF-κB signaling activation were accompanied by HO-1 upregulation in mice with ALI [29-31]. These findings suggested that HO-1 upregulation could ameliorate airway inflammation in ALI. Herein, treatment with LOLE significantly upregulated HO-1 in RAW264.7 cells and mouse, indicating that the promotive effect of LOLE on HO-1 could exert a protective effect in ALI.

Cumulative evidence has shown that natural plant extracts have anti-inflammatory effects on ALI [3, 32, 33]. These effects are triggered by the reduction of inflammatory cell recruitment, the secretion of specific molecules, MAPK/NF-κB activation, and HO-1 upregulation. A previous in vitro study [19] and the current in vivo results revealed that LOLE could suppress inflammatory molecules and MAPK/NF-κB activation. In addition, LOLE could upregulate the expression of HO-1 in both mice and macrophages. We thus hypothesized that the inhibitory effect of LOLE could be related to MAPK/NF-κB inactivation and HO-1 induction. Therefore, these results revealed the protective effect and the underlying mechanism of LOLE in LPS-induced ALI, indicating that LOLE may prove useful as a potential adjuvant therapy in ALI.
